# Medication-related problems in critical care survivors: a systematic review

**DOI:** 10.1136/ejhpharm-2023-003715

**Published:** 2023-05-04

**Authors:** Abigail Short, Joanne McPeake, Mark Andonovic, Stuart McFee, Tara Quasim, Alastair Leyland, Martin Shaw, Theodore Iwashyna, Pamela MacTavish

**Affiliations:** 1 NHS Forth Valley, Stirling, Stirling, UK; 2 The Healthcare Improvement Studies Institute, University of Cambridge, Cambridge, UK; 3 School of Medicine, Dentistry and Nursing, University of Glasgow, Glasgow, UK; 4 NHS Greater Glasgow and Clyde, Glasgow, UK; 5 MRC/CSO Social and Public Health Sciences Unit, University of Glasgow, Glasgow, UK; 6 Johns Hopkins University, Baltimore, Maryland, USA

**Keywords:** Critical Care, CLINICAL MEDICINE, PHYSICAL AND REHABILITATION MEDICINE, Quality of Health Care, CRITICAL CARE

## Abstract

**Objectives:**

There are numerous, often single centre discussions of assorted medication-related problems after hospital discharge in patients who survive critical illness. However, there has been little synthesis of the incidence of medication-related problems, the classes of medications most often studied, the factors that are associated with greater patient risk of such problems or interventions that can prevent them.

**Methods:**

We undertook a systematic review to understand medication management and medication problems in critical care survivors in the hospital discharge period. We searched OVID Medline, Embase, PsychINFO, CINAHL and the Cochrane database (2001–2022). Two reviewers independently screened publications to identify studies that examined medication management at hospital discharge or thereafter in critical care survivors. We included randomised and non-randomised studies. We extracted data independently and in duplicate. Data extracted included medication type, medication-related problems and frequency of medication issues, alongside demographics such as study setting. Cohort study quality was assessed using the Newcastle Ottowa Score checklist. Data were analysed across medication categories.

**Results:**

The database search initially retrieved 1180 studies; following the removal of duplicates and studies which did not fit the inclusion criteria, 47 papers were included. The quality of studies included varied. The outcomes measured and the timepoints at which data were captured also varied, which impacted the quality of data synthesis. Across the studies included, we found that as many as 80% of critically ill patients experienced medication-related problems in the posthospital discharge period. These issues included inappropriate continuation of newly prescribed drugs such as antipsychotics, gastrointestinal prophylaxis and analgesic medications, as well as inappropriate discontinuation of chronic disease medications, such as secondary prevention cardiac drugs.

**Conclusions:**

Following critical illness, a high proportion of patients experience problems with their medications. These changes were present across multiple health systems. Further research is required to understand optimal medicine management across the full recovery trajectory of critical illness.

**PROSPERO registration number:**

CRD42021255975.

**WHAT IS ALREADY KNOWN ABOUT THIS TOPIC**

- Survivors of critical illness experience multiple transitions of care following critical care discharge. As a result, there can be subsequent interruptions and disruptions with medication management.

**WHAT THIS STUDY ADDS**

- As many as 80% of critical care survivors can experience problems with medication management during recovery. This includes unintentional discontinuation of chronic disease medications in up to a quarter of patients; conversely, many patients are continued, often inappropriately, on acutely prescribed drugs.

**HOW MIGHT THIS STUDY MIGHT AFFECT RESEARCH, PRACTICE OR POLICY**

- Further research is required to understand optimal medicine management across the full recovery trajectory of critical illness.

## BACKGROUND

Patients admitted to critical care can often experience both rapidly changing organ dysfunctions and multiple transitions of care.^1^ Both factors place patients at risk for disruptions of medications.^2 3^ Medication regimens at hospital discharge can differ from preadmission medications, and when these changes are not appropriately managed across transitions of care, it can lead to subsequent patient harm.^4–6^

There are few standard guidelines as to how to prevent or detect-and-remediate afterwards, such disruptions in care. At present there are no published systematic reviews which attempt to synthesise key questions such as: how common are such medication-related problems after critical illness? Which medication classes are involved in these problems? Are some patients at greater risk? Moreover, there is no evidence summary examining what can be done to avoid patient harm from medication-related problems after a stay in the intensive care unit (ICU).

Therefore, we undertook a systematic review to understand medication management and medication problems in critical care survivors in the hospital discharge period. We hypothesised that there would be a broad spectrum of medication issues and challenges for survivors.

## METHODS

### Search strategy

The PRISMA (Preferred Reporting Items for Systematic Reviews and Meta-Analyses) checklist was followed for reporting this systematic review.^7^ The review protocol was registered with PROSPERO (CRD 42021255975).

PROSPERO and the Cochrane database were searched to ensure that no other systematic review was underway. One review was underway; this review aimed to examine the impact of a medication-related interventions during transitions of care between ICU settings and the general ward environment.^8^ No review was examining the transition from hospital to home, or longer-term medication management in critical care survivors.

We electronically searched OVID Medline, EMBASE, PsychINFO, CINAHL and the Cochrane database. Our search took place on 20 July 2021, with an update undertaken on 31 August 2022. The search strategy, which was supported by an experienced librarian (SM), cross-referenced medicine management and critical illness with appropriate keywords and subject headings (see online supplemental file S1 for full details). The search was limited to research published between 2001 and 2022. This date restriction was applied as the importance of medicine safety and adverse event reporting was highlighted via the Institute of Medicine’s seminal ‘To Err is Human*’* white paper in 2000, with subsequent widespread implementation of changes to medicines reconciliation internationally.^9^

10.1136/ejhpharm-2023-003715.supp1 Supplementary data

### Study selection

The research question was generated via the participants, interventions, comparisons and outcomes (PICO) model (table 1).^10^

**Table 1:** PICO model alongside design inclusion/exclusion criteria

Characteristics	Inclusion	Exclusion
Participants	Patients admitted to critical care	Patients without a critical care encounter
Interventions	Any as per definition	Studies that did not provide data on medicines at hospital discharge or in the posthospital discharge period
Comparisons	N/A	N/A
Outcomes	Medicine review at hospital discharge or in the posthospital discharge period	Medicine review in ICU or in hospital only
Study design	Randomised, quasi-experimental (parallel control group trials and pre/postintervention trials) observational	Case reports, reviews, editorials, quality improvement studies with interventions, theses or other commentaries

ICU, intensive care unit; N/A, not available; PICO, participant, intervention, comparison and outcomes.

We included articles which met the following criteria: involving adults (admitted to an adult critical care environment) and including medicine management data at hospital discharge or following hospital discharge. Thus, we excluded studies which examined transitions of care in the hospital environment, or those which reported in-hospital medicine management in isolation. We also excluded studies which were not peer reviewed, were non-English language, included quality improvement interventions or were in abstract format only.

Each citation was independently reviewed for eligibility by two clinicians via a review of title and abstract, and then, where appropriate, full-text articles. All extracted papers also had their reference list hand-searched to ensure that all relevant papers had been included. AS, JM, SM, MA and PM undertook this process. Disagreement was resolved via regular meetings of this study team.

### Quality assessment

Cohort study quality was assessed using the Newcastle Ottowa Score checklist.^11^ This consists of the three main domains to assess the quality and risk of bias. These are: patient selection (cohort data source, representativeness and ascertainment of exposure to the outcome of interest), comparability of cohort and outcome assessment (including adequate follow-up time, acquisition of outcome and adequacy of follow-up). Data on risk of bias and the overall quality assessment can be found in online supplemental file S2.

10.1136/ejhpharm-2023-003715.supp2 Supplementary data

### Data extraction

Data were extracted by four clinicians (AS, JM, SM and PM) and entered into a standardised template. All data were cross-checked by a second reviewer. Any discrepancies were resolved by consensus. Data extracted included author, date, location, study design, population, number of patients studied, gender, age, time of follow-up, type of medicine reviewed and main study outcomes. Outcomes were chosen a priori, and the template was piloted before implementation.

Studies included in this review often examined different classes of medicines. For example, some examined analgesics and others cardiovascular drug management. We placed no restrictions on the type of drug examined as we were interested in obtaining a global view of medicine management.

## RESULTS

### Study selection

**Figure 1:**
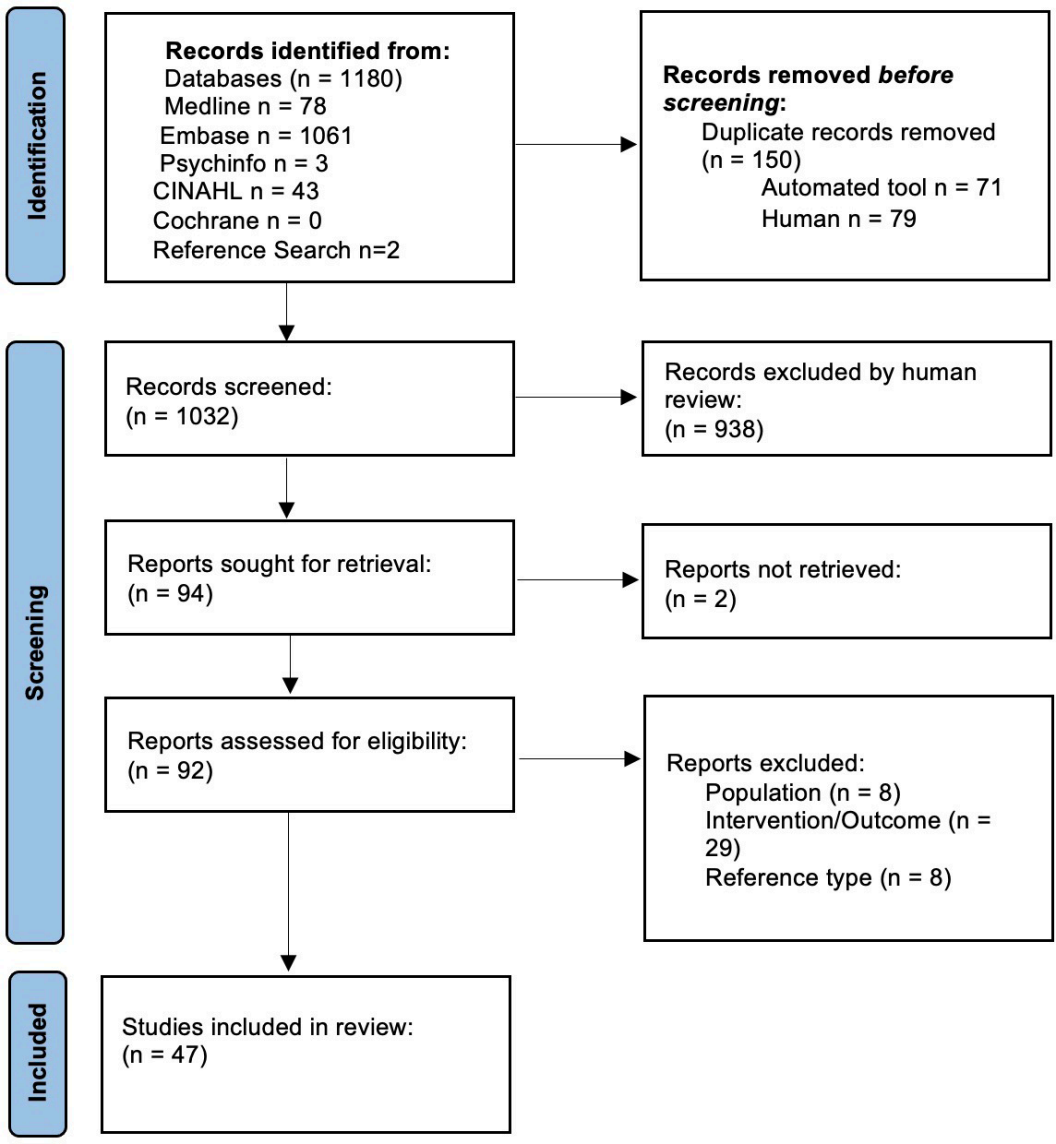
PRISMA flowchart.

The database search initially retrieved 1180 studies; following the removal of duplicates, 1032 unique studies were identified for title and abstract review. A total of 938 studies were excluded as they did not meet the study inclusion criteria, leaving 94 full-text articles for review; 47 studies were then excluded during the full-text review, resulting in 45 papers meeting review inclusion (figure 1). Of note, two papers had the same population for analysis. However, both analyses examined different elements of care which were of interest, and so both were included.^12 13^

### Summary of studies

Studies varied widely in their size, scope and methodology. Across the 47 included studies: 28 (60%) were from the USA, six (13%) from Canada, five (11%) from the UK, two (4%) from Australia and one (2%) each from Brazil, Belgium, Denmark, South Korea, Sweden and Switzerland. In total, 29 studies (62%) described medicine management at hospital discharge, and the remaining 18 studies (38%) examined medicine management in the posthospital discharge period. The full characteristics of included studies are presented in the online supplemental file S3. A summary of the main features of the research included is presented in table 2. Due to the heterogeneity of the study outcomes and the data captured, we were unable to undertake a meta-analysis.

10.1136/ejhpharm-2023-003715.supp3 Supplementary data

10.1136/ejhpharm-2023-003715.supp4 Supplementary data

10.1136/ejhpharm-2023-003715.supp5 Supplementary data

10.1136/ejhpharm-2023-003715.supp6 Supplementary data

10.1136/ejhpharm-2023-003715.supp7 Supplementary data

**Table 2:** Overview of studies included in this review

Study characteristic (n=47)	N (%)
Geographical region	
Australia	2 (4)
Belgium	1 (2)
Brazil	1 (2)
Canada	6 (13)
Denmark	1 (2)
South Korea	1 (2)
Sweden	1 (2)
Switzerland	1 (2)
UK	5 (11)
USA	28 (60)
Study type	
Retrospective observational cohort	40 (85)
Prospective observational cohort	7 (15)
Study scope	
Multicentre	12 (26)
Single centre	35 (74)
Study population focus	
Mixed (general/mixed medical and surgical specialties)	29 (62)
Medical ICU (MICU)	6 (13)
Cardiac ICU (CICU)	1 (2)
Trauma/surgical/neurosurgical ICU	4 (9)
Elderly patient/chronic medications	5 (11)
Cancer centre general ICU	1 (2)
Acute kidney injury/renal replacement need	1 (2)
Medication type studied*	
Opiates/analgesics	12
Gastroprotection/acid suppressants	12
Psychotropic	14
Medication changes/PIMs/AIMs	11
Cardiovascular	4
Main timepoint examined	
Hospital discharge	29 (62)
Hospital discharge and post-discharge follow-up	5 (11)
Outpatient follow-up <6 months	8 (17)
Outpatient follow-up >6 to <9 months	2 (4)
Outpatient follow-up to >9 months to 1 year	2 (4)
Outpatient follow-up >1 year	1 (2)
Source of study data	
Clinical record including discharge prescription/summary	32 (68)
Face-to-face interview	7 (15)
National databases	8 (17)

*n=53 as some papers studied more than one medication class.

AIM, actually inappropriate medication; ICU, intensive care unit; PIM, potentially inappropriate medication.

### Risk of bias

The quality assessment for the included studies is shown in online supplemental file S2. The overall quality of the studies varied. Across all 47 studies included, the median (IQR) Newcastle Ottawa score was 7 (5–8) for the studies included.

### Medication-related problems

#### Overview of included studies

Eleven studies described medication-related problems in critical care survivors^5 12–21^ (online supplemental file S3, table 1). Two papers examined medication-related problems in the same population of older ICU survivors in the US.^12 13^ Both studies were included as they focused on different elements of care. Across the studies there was a wide range of definitions and classifications used to define medication-related problems (table 3). Four studies examined medication-related problems at hospital discharge,^12 13 15 18^ and the remaining seven studies examined medication-related problems within the ICU follow-up setting.^5 14 16 17 19–21^ One study specifically examined the outcomes of patients admitted to critical care for COVID-19.^20^

**Table 3:** Terminology and definition of medication-related problems used across included studies

Terminology to define a medication-related problem	Definition	Study
Inappropriate medication Discontinuation	Unintentional discontinuation of chronic medications (eg, a statin or antiplatelet/anticoagulant)	Bell *et al* (2006)^22^
Discrepancy: the need for a pharmacy intervention	Interventions included dose adjustments, additional therapy, inappropriate therapy discontinued and patient/family counselling	Bottom-Tanzer *et al* (2020)^16^
PIMs AIMs	Beer criteria^63^ For example, stress ulcer prophylaxis which should have been discontinued at ICU/hospital discharge	Morandi *et al* (2011)^12^ Morandi *et al* (2013)^13^ Galli *et al* 2016^18^
Medication-related problem	Included drug omissions, drug adjustments, duration of treatment advice; patient education and counselling (eg, re-titration of preadmission gabapentin for neuropathic pain)	MacTavish *et al* (2019)^5^ MacTavish *et al* (2020)^19^
Pharmacist intervention	Included drug omissions, drug adjustments, adverse drug event identified or prevented, duration of treatment advice; patient education and counselling (eg, identification of adverse drug events such as hypoglycaemia)	Stollings *et al* (2018)^21^
Pharmacist intervention	Included GDMT optimisation, refill assistance, medication cost assistance, pill box provision, lab monitoring, medication cessation, medication addition and medication dose adjustment (eg, GDMT optimisation of heart failure drug treatment)	Adie *et al* (2021)^14^
Medication changes	Classified as appropriate or inappropriate based on discussion with clinical team, patient and ongoing clinical indication (eg, inappropriate continuation of anticoagulants)	MacTavish *et al* (2021)^20^
Potential medication errors and medicine-related problem	Included Inappropriate discontinuation of chronic medications, difficulties obtaining supplies, administration, information and understanding of the suitability of prescriptions (eg, inappropriate continuation of sedatives at hospital discharge)	Eijsbroek *et al* (2013)^17^

AIM, actually inappropriate medication; GDMT, guideline-directed medical therapy; ICU, intensive care unit; PIM, potentially inappropriate medication.

#### Observations

There was variation in the number of patients impacted by medication-related problems. For example, a multicentre Canadian study of 834 patients reported that a third of patients were impacted by a medication-related problem at hospital discharge,^15^ whereas in in a single centre study of older adults in the US, 85% of patients experienced a medication-related problem (defined as a potentially inappropriate medication).^13^ In the ICU follow-up setting, 62.80–100% of patients who attended experienced a medication-related problem or required a pharmacy intervention.^5 14 19 21^ These medication-related problems were classified as clinically significant in 86.40% of medication-related problems examined in one study, and 64% in a further single centre study.^5 19^

The studies varied in the types of drugs involved in medication-related problems. One study examined the proportion of patients unintentionally discharged from hospital with chronic medication omitted and found that approximately one-third of patients experienced problems with their chronic disease management medication.^15^ A further study undertaken in the ICU follow-up setting found that 5.30% of patients did not have medications for chronic conditions restarted following discharge from hospital.^17^ Two studies, one at hospital discharge and one based in the ICU follow-up setting, found that analgesics including opiates were the most likely medications to be involved in medication-related problems.^5 12^

#### Risk factors

Risk factors for medication-related problems at hospital discharge included the omission of medications at ICU discharge,^15^ not being discharged home^12^ and discharge from a surgical service.^12^ Risk factors for medication-related problems in the ICU follow-up setting included hospital length of stay, and the number of ICU discharge medications and analgesic requirements at ICU discharge and at the clinic attendance.^5 19^

### Gastrointestinal protection medications

#### Overview of included studies

There were 12 studies which examined the use of gastrointestinal protection agents in the hospital discharge period (online supplemental file S3, table 2).^17 22–32^ This drug group includes proton pump inhibitors (PPI), H2 receptor antagonists and Sucralfate. Ten studies examined gastrointestinal protection use at hospital discharge,^23–32^ one examined dispensing of gastric acid suppressors up to 90 days following discharge^22^ and three studies examined gastrointestinal protection in the outpatient follow-up setting.^17 29 30^

#### Observations

The number of inappropriate gastrointestinal protection agent prescriptions reported at hospital discharge ranged considerably across the studies included. For example, in three studies, all gastrointestinal protection agents prescribed at hospital discharge were deemed to be prescribed inappropriately.^23 30 32^ In contrast, a single centre study from the US found that only 15.7% of prescriptions at hospital discharge were inappropriate.^27^ Three studies examined inappropriate continuation of these drugs beyond hospital discharge.^23 29 30^ In the first, 58.20% of patients prescribed gastrointestinal protection at 3 months were prescribed them inappropriately.^30^ The second study, which examined gastrointestinal protection at a 4-week telephone follow-up call, found that only 5% of patients prescribed gastrointestinal protection had a compelling reason for continuation.^29^ In the most recent study, 64% of patients remained on proton pump inhibitors with no indication following hospital discharge.^23^ Conversely, three studies highlighted that up to 15.40% of patients were not restarted on previously prescribed (prehospital) gastrointestinal protection at hospital discharge.^22 24 30^

#### Risk factors

Several risk factors for continued use of gastrointestinal protection medications were identified across three studies. Risk factors included discharge to a long-term care facility, an ICU admission a surgical (as opposed to medical) admission and mechanical ventilation.^22 23 25^

### Psychotropic medications

#### Overview of included studies

Fourteen studies examined the use of psychotropic medications in survivors of critical care (online supplemental file S3, table 3).^33–46^ Drugs included were antipsychotic and anxiolytic agents alongside antidepressants. Twelve studies examined psychotropic medications use in critical care survivors at hospital discharge;^34–45^ one study examined their use up to 180 days following hospital discharge^33^ and the final study examined the use of these medications up to 1 year posthospital discharge.^46^

#### Observations

Across the studies, in those patients prescribed psychotropic medications during admission, there was wide variation in the continued use of these drugs at hospital discharge (range 10.30–61%), although the appropriateness of this continued use was difficult to assess across the studies.^34–45^ Four studies gave details on the prescription appropriateness at hospital discharge; one single centre US study found 54 of their cohort of 161 patients (34%) had been continued on antipsychotics or anxiolytics at hospital discharge, with no patient having a documented reason for their use.^37^ In another single site study, 68.40% of patients prescribed atypical antipsychotics at hospital discharge had no ongoing indication for their use,^39^ while a further study found that 24.40% of survivors treated with antipsychotics during critical illness, remined on these medicine at hospital discharge, despite two-thirds of these survivors having normal mental status documented.^45^ Finally, a single centre cohort study based in a trauma and neurosurgical unit found that 67.10% of prescriptions, the majority of which were for quetiapine, were inappropriate at hospital discharge.^44^

#### Risk factors

One large Danish registry study examined the use of antipsychotics in mechanically ventilated patients, in comparison with a hospitalised and a general population cohort, up to 1 year posthospital discharge. In this study, they found that in those who had received mechanical ventilation, the risk of new psychoactive medication prescriptions increased in the first 3 months following hospital discharge in comparison with the hospital and general control population, although these differences had largely resolved by 12 months.^46^ Another VA registry study in the US also found that patients with a diagnosis of sepsis were more likely to be continued on antipsychotics in the posthospital discharge phase (up to 180 days).^45^ Seven studies found critical illness specific variables, such as ICU length of stay, severity of illness and the type of admission were risk factors for the continuation of psychotropic medications.^34 35 38 40–42 44^

### Analgesia

#### Overview of included studies

In total, 12 studies examined the use of analgesics in the posthospital discharge period (online supplemental file S3, table 4).^5 17 20 47–55^ Analgesics examined included simple analgesics such as paracetamol alongside weak and strong opioids. Five studies examined the use of analgesia at hospital discharge,^47 48 50 52 55^ three studies were based in the ICU follow-up setting^5 17 20^ and six studies examined analgesics longitudinally across the critical care recovery period (up to 24 months following discharge.^48 49 51 53–55^ Studies varied in their inclusion definition, with some studies examining chronic opioid use, some examining the outcomes of opioid naïve patients and others examining analgesics in all ICU survivors.

#### Observations

New analgesic prescribing was reported in 10 studies.^5 17 20 47 48 50–52 54 55^ Five studies reported new analgesia prescriptions at hospital discharge (number of patients receiving new analgesics at hospital discharge (range 31.80–47.10%).^47 48 50 52 55^ Three studies were set in the ICU follow-up setting, and two of the studies took place with the general ICU population and reported new analgesia requirements in 27% and 76% of patients included.^5 17^ In one of these studies, 16% of the total patient cohort (183 patients) were receiving new opiate prescriptions in the post-ICU recovery phase.^5^ A further study, also in the ICU follow-up setting, specifically examined new analgesic requirements in critically ill COVID-19 survivors and found a significant increase in the number of patients taking regular analgesia following severe COVID-19 infection.^20^ The final study which reported increased analgesic use, demonstrated that 20% of patients filled new opiate prescriptions within 7 days of hospital discharge; however, persistent opiate use at 1 year fell to between 2.60–4.90%.

Conversely, a large population-based cohort study of elderly ICU survivors with chronic opioid use found relatively static opioid use in the posthospital discharge period (up to 180 days posthospital discharge), with 22% of patients on a higher dose of opiate compared with prehospitalisation, 19.80% receiving the same dose, and 21.50% of patients receiving a lower dose.^49^

#### Risk factors

Nine studies reported risk factors for continuation of analgesics.^47–52 54 55^ Risk factors included a cardiac critical care and surgical admissions, a history of illicit drug use (including alcohol and substance use disorders), intubation, younger age, a diagnosis of sepsis, previous benzodiazepine use, chronic opiate use preadmission, a higher cumulative dose of opiate in the ICU and an ICU admission diagnosis of malignancy.

### Cardiac medications

#### Overview of included studies

In total, four studies examined cardiac medication management in survivors of critical care (online supplemental file S3, table 5).^22 33 56 57^ Two studies examined cardiac medication use at hospital discharge^56 57^ and two studies examined their use following hospital discharge.^22 33^

#### Observations

One study found that 34% of patients were continued on midodrine at hospital discharge, with an estimated 50% of these prescriptions deemed inappropriate.^57^ In the two studies which examined cardiac medications in the posthospital discharge period, between 15.10–22.80% of critical care and sepsis survivors did not have chronic cardiac medications such as statins and antiplatelets restarted or refilled.^22 33^

#### Risk factors

An ICU admission and a diagnosis of sepsis were risk factors for unintentional discontinuation of chronic cardiac medications.^22 33^

## DISCUSSION

This systematic review aimed to understand changes to medication management and medication problems in critical care survivors during the hospital discharge period. It has found that as many as 80% of critical care survivors can experience problems with medication management during recovery.^13 19^ This included unintentional discontinuation of chronic disease medications in up to a quarter of patients; conversely, we also found that many patients were continued, often inappropriately, on acutely prescribed drugs. Problems occurred across multiple classes of drugs, including gastrointestinal protection, psychotropic, analgesic and cardiac medications. Several risk factors for medication-related problems emerged including the need for mechanical ventilation, a sepsis diagnosis and a critical care admission (vs hospital admission only).

It is well recognised that pharmacists play a key role within the ICU setting. However, at present their role in recovery programmes is sporadic, and has not been integrated into national recommendations. For example, the UK Faculty of Intensive Care Medicine’s Guidelines for the provision of intensive care services makes no mention of the pharmacist in the delivery of ICU after care.^58^ The findings of this review would suggest that the role of the pharmacist within recovery programmes and across the recovery timeline more broadly may improve outcomes for patients, and potentially provide benefits to the healthcare system. More work in this area is required.

This review found inconsistent results in relation to pain management and opiate use in the posthospital discharge period. Studies demonstrated both relatively static use, as well as significant increases in opiate prescribing,^20 51^ although synthesis of these data was hindered by the heterogeneity of the inclusion criteria across studies. It is important to recognise that no study matched medication management and medication-related problems with patient reported outcomes, such as global quality of life or pain scores. We do not know if patients were discontinued opiates with the addition of subsequent substitutes, or if these drugs were inappropriately prescribed. Recent evidence has demonstrated chronic pain occurs in up to two-thirds of ICU survivors; as such, future research should link patient-reported outcomes (eg, pain scores with medicine management), thus ensuring a holistic picture of the challenges which patients face following hospital discharge.^59 60^

We were unable to undertake a meta-analysis with these data owing to the heterogenous nature of the research available. Studies varied in the definition of medication-related problems and examined these issues across various timeframes. Given this, future work should examine which outcomes are important to gather and the timeframe which is most appropriate. This step would allow data to be fully synthesised and relevant recommendations for practice to be established.

There are limitations to this review. First, we were unable to examine patient-level factors which could have contributed to the medication-related problems described across transitions of care. Socioeconomic factors such as educational attainment and access to adequate financial support are known to influence recovery from critical illness.^61 62^ These social factors may have influenced issues such as medication-related problems and access to drugs within specific healthcare systems. Future work should examine patient demographics, alongside healthcare access following discharge, to understand any individual risk factors. Second, many of the studies included used large-scale national databases to examine outcomes. These databases have inherent problems; most notably in this review, the inability to understand why medicines may have been stopped or started. Third, there was a wide range of cohort sizes included in this review. There was also significant variation in the timepoint of measure, the type of measure and the setting in which the measure was undertaken. The event rate of medication errors was also inconsistently reported; having this event rate would have allowed an enhanced understanding of the interaction between the sample size and the outcomes described. This variation makes it challenging to compare studies and synthesise them accurately. Future work should endeavour to create a standardised approach to outcome measurement in this field.

## Conclusions

Following critical illness, patients can experience problems and changes to medicines. These changes are present across multiple health systems and classes of medications. Further research is required to understand optimal medicine management across the full recovery trajectory of critical illness.

## Data Availability

All data relevant to the study are included in the article or uploaded as supplementary information. All data relevant to the study is included in the article or uploaded as supplementary information.
