# Supplementary material for: Medication-related problems in critical care survivors: a systematic review
Source: Eur J Hosp Pharm. 2023 May 4;30(5):250–6. doi: 10.1136/ejhpharm-2023-003715 (PMC10447966; doi:10.1136/ejhpharm-2023-003715)
Supplement: Supplementary data [file ejhpharm-2023-003715supp001.pdf]

Date stamp – 31st May 2022

OVID databases: Medline, EMBASE, PsycInfo,

Search criteria:

1. (medication adj2 error*).mp.
2. (medication adj2 problem*).mp.
3. exp Medication Errors/
4. exp "Drug-Related Side Effects and Adverse Reactions"/ or exp Medication Errors/
5. exp Drug Prescri*/
6. (medication* adj2 intervention*).mp.
7. (prescri* adj2 error*).mp.
8. (prescri* adj2 intervention*).mp.
9. 7 or 8
10. 1 or 2 or 3 or 4 or 5 or 6
11. exp Medication Errors/ or medicine reconciliation.mp. or exp Drug Prescri*/ or exp Medication Reconciliation/
12. (inappropriate adj2 medication*).mp.
13. 9 or 10 or 11 or 12
14. exp Critical Care/
15. intensive care.mp.
16. critical care.mp.
17. 14 or 15 or 16
18. (hospital adj2 discharge).mp.
19. exp Patient Discharge/
20. (post adj2 discharge).mp.
21. (intensive adj2 care adj2 survivor*).mp.
22. exp Survivors/
23. 18 or 19 or 20 or 21 or 22
24. 13 and 17
25. 23 and 24
26. 17 and 23
27. exp Antipsychotic Agents/ or antipsych*.mp.
28. opi*.mp.
29. gastroprotect*.mp.
30. Histamine H2 Antagonists/ or Proton Pump Inhibitors/ or stress ulcer prophylaxis.mp. or Anti-Ulcer Agents/
31. acid suppress* therapy.mp.
32. 29 or 30 or 31
33. 26 and 27
34. 26 and 28
35. 26 and 32

EBSCO – CINAHL

| | |
|---|---|
| S22 | S12 AND S15 AND S21 |
| S21 | S16 OR S17 OR S18 OR S19 OR S20 |
| S20 | survivor* |
| S19 | TX intensive n2 care n2 surviv* |
| S18 | TX post n2 discharge* |
| S17 | patient discharge |
| S16 | TX hosp* n2 discharge |
| S15 | S13 OR S14 |
| S14 | TX intensive care |
| S13 | critical care |

| | |
|---|---|
| S12 | S1 OR S2 OR S3 OR S4 OR S5 OR S6 OR S7 OR S8 OR S9 OR S10 OR S11 |
| S11 | TX inappropriate* n2 medic* |
| S10 | medication reconciliation |
| S9 | medic* reconciliation |
| S8 | TX unintention* n2 medic* |
| S7 | TX prescri* n2 intervention* |
| S6 | TX prescri* n2 error* |
| S5 | TX medic* n2 intervention* |
| S4 | drug prescription* |
| S3 | medication error |
| S2 | TX medic* n2 problem* |
| S1 | TX medic* n2 error* |
