# Supplementary material for: Medication-related problems in critical care survivors: a systematic review
Source: Eur J Hosp Pharm. 2023 May 4;30(5):250–6. doi: 10.1136/ejhpharm-2023-003715 (PMC10447966; doi:10.1136/ejhpharm-2023-003715)
Supplement: Supplementary data [file ejhpharm-2023-003715supp002.pdf]

Table S2: Quality Assessment

Author/Year	Design	Selection	Comparability	Outcome	Total
Academia et al (2020)	Observational cohort	3	1	3	**7**
Adie et al (2021)	Observational cohort	2	0	2	**4**
Bell et al (2006)	Observational cohort	4	2	3	**9**
Bell et al (2011)	Observational cohort	4	2	3	**9**
Blackett et al (2021)	Observational cohort	3	2	3	**8**
Bottom-Tanzer et al (2021)	Observational cohort	2	0	3	**5**
Choon et al (2021)	Observational cohort	3	1	3	**7**
Coe et al (2020)	Observational cohort	3	2	3	**8**
Dixit et al (2021)	Observational cohort	3	2	3	**8**
Eijsbroek et al (2013)	Observational cohort	2	0	1	**3**
Farley et al (2013)	Observational cohort	3	0	3	**6**
Farrell et al (2010)	Observational cohort	4	2	3	**9**
Farrokh et al (2017)	Observational cohort	3	0	2	**5**
Flurie et al (2015)	Observational cohort	2	0	3	**5**
Franchitti et al (2020)	Observational cohort	3	0	3	**6**
Galli et al (2016)	Observational cohort	2	2	2	**6**
Gilbert et al (2017)	Observational cohort	3	1	3	**7**
Hatch et al (2010)	Observational cohort	1	0	2	**3**
Jasiak et al (2013)	Observational cohort	2	0	3	**5**
Karamchandani, Schoaps et al (2019)	Observational cohort	3	0	3	**6**
Karamchandani, Pyati et al (2019)	Observational cohort	3	0	2	**5**
Kram et al (2015)	Observational cohort	3	0	3	**6**
Krancevich et al (2022)	Observational cohort	3	2	3	**8**
Lambert et al (2021)	Observational cohort	3	1	2	**6**
Levine et al (2019)	Observational cohort	3	0	3	**6**
MacTavish et al (2019)	Observational cohort	3	2	3	**8**
MacTavish et al (2020)	Observational cohort	4	2	3	**9**
MacTavish et al (2021)	Observational cohort	3	1	3	**7**
Marshall et al (2016)	Observational cohort	4	2	3	**9**

Mehta et al (2020)	Observational cohort	3	0	2	**5**
Morandi et al (2013)	Observational cohort	3	2	3	**8**
Morandi et al (2011)	Observational cohort	3	0	3	**6**
Murphy et al (2008)	Observational cohort	2	1	2	**5**
Rizvi et al (2019)	Observational cohort	4	2	3	**9**
Rowe et al (2015)	Observational cohort	2	2	3	**7**
Shin (2015)	Observational cohort	4	0	3	**7**
Stollings et al (2018)	Observational cohort	2	0	3	**5**
Tan et al (2016)	Observational cohort	3	0	3	**6**
Tollinche et al (2022)	Observational cohort	4	2	3	**9**
Tomichek et al (2016)	Observational cohort	3	2	3	**8**
Von Oelreich et al (2021)	Observational cohort	4	2	3	**9**
Wang et al (2018)	Observational cohort	3	1	3	**7**
Witcraft et al (2021)	Observational cohort	2	0	3	**5**
Wolht et al (2007)	Observational cohort	3	0	2	**5**
Wunsch et al (2014)	Observational cohort	4	2	3	**9**
Wunsch et al (2020)	Observational cohort	4	2	3	**9**
Yaffe et al (2017)	Observational cohort	3	2	3	**8**
