# Supplementary material for: Medication-related problems in critical care survivors: a systematic review
Source: Eur J Hosp Pharm. 2023 May 4;30(5):250–6. doi: 10.1136/ejhpharm-2023-003715 (PMC10447966; doi:10.1136/ejhpharm-2023-003715)
Supplement: Supplementary data [file ejhpharm-2023-003715supp003.pdf]

S3: Table 1 POTENTIAL INAPPROPRIATE MEDICATION AND ACTUAL INAPPROPRIATE MEDICATION DATE EXTRACTION TABLE

Author	Yr	Country	ICU Population	Nature	Timeline	n	Gender	Age	Medication type	Results
Adie et al.	2021	USA	Cardiac	Single centre<br><br>Retrospective, observational cohort study	Post-discharge CICU clinic: 2-week post discharge	106 (70)	M-71%	Median 65 (54 – 72)	Any	**Issues encountered:** Results described as n=number of interventions. Median number of pharmacist interventions = 4 (3-5), each patient had at least 1 intervention. Number of drugs requiring a dose adjustment (n=46), optimisation (n=42), change (n=18), addition (n=23), cessation (n=21). Median number of medication changes = 2 (1-3).<br>Pillbox provision (n=8) and refill assistance (n=16).<br>**Risk factors for problems:** Nil described<br>**Protective factors against problems:** Nil described
Bell et al	2006	Canada	General	Multicentre<br><br>Retrospective observational cohort study	Hospital discharge	834	M-57%	Median 65 (IQR 50-76)	Statins<br>Antiplatelet and anticoagulant<br>L-thyroxine<br>Regular inhalers<br>AST<br>Allopurinol	**Issues encountered:** Medication discontinuation at discharge – n = 251/834 (33%) with 1 or more medications affected<br>**Risk factors for problems**: Medications omitted at ICU discharge higher OR for omission at hospital discharge.<br>**Protective factors against problems:** Admission to academic ICU, admission with medical diagnosis
Bottom-Tanzer et al	2020	USA	Trauma Medical	Single Centre<br><br>Prospective observational cohort study	ICU-clinic:<br>2-, 12-, and 24-weeks post hospital discharge	70	M-59%	Mean 54.7 (SD 15.7)	Any	**Issues encountered:** n = 106/118 (94.6%) pharmacy reviews had one or more discrepancy.<br>116 interventions – dose adjustments (n = 19), additional therapy (n = 23), inappropriate therapy discontinued (n=27), patient/family counselling (n=47).<br>**Risk factors for problems:** Nil described<br>**Protective factors against problems:** Time from hospital admission: reduced average number of medication interventions at 24-week visit (0.8/pt) vs 2-week visit (1.2/pt)
Eijsbroek et al	2013	UK	General	Single Centre<br><br>Retrospective observational cohort study	ICU-clinic:<br>3-, to 9-months post hospital discharge	21	M-53%	Mean 64.4 (SD 13)	Any	**Issues encountered:** Discontinuation of chronic medications and not restarted – 5.3%.<br>Physical issues with medication management and comprehension issues with medications amongst patients and carers<br>**Risk factors for problems:** Nil described<br>**Protective factors against problems:** Nil described
Galli et al	2016	Brazil	Medical Cardiac<br><br>Patients >60yrs old	Single centre<br><br>Retrospective observational cross-sectional	Hospital discharge	486	M-55%	Median 71 (IQR 65-77)	Any	**Issues encountered:** n = 74/1864 (3.9%) PIMs identified in population during hospitalisation continued at hospital discharge. 41.1% of PIMs at discharge were medications initiated in ICU.<br>**Risk factors for problems:** Unclear which specific to

				study						medications at discharge.<br>**Protective factors against problems:** Nil described
MacTavish et al.	2019	UK	General	Single Centre.<br><br>Retrospective observational cohort study	ICU-clinic:<br>6- weeks to 3-years post hospital discharge	47	M-66%	Median 52 (IQR 44-57)	Any	**Issues encountered:** n = 38/47 medication-related problem:<br>Drug omissions – 20/47 (29%)<br>Dose adjustment 13/47 (19%)<br>Duration of treatment advice 12/47 (17%)<br>Patient unaware of medication change – 26/47 (55%)<br>Patient expressed concerns re medications – 28/47 (60%)<br>69 (18.6%) medications had problems at review, 44/69 (64%) classified with severity score >/= 3.<br>**Risk factors for problems:** Number of pain medications at ICU discharge. Nil other variables significant.<br>**Protective factors against problems:** Nil described
MacTavish et al.	2020	UK	Medical Surgical	Multicentre<br><br>Prospective observational cohort study	ICU-clinic:<br>4- to 12- weeks post hospital discharge	183	M-56%	Median 58 (IQR 50-65)	Any	**Issues encountered:** Medication related problems = 198, Medication omissions – n = 27/198.<br>Severity of MRP: Minor 27/198, Moderate 141/198, Severe 30/198.<br>Medications most affected: Analgesia > CVS > GI > Neuroleptic<br>**Risk factors for problems:** Hospital LOS, number of ICU discharge medications, and prescription of analgesia on WHO Step 2 classification.<br>**Protective factors against problems:** Nil described
MacTavish et al.	2021	UK	General (COVID-19 survivors)	Single centre<br><br>Prospective observational cohort study	3-7 months post hospital discharge.<br>Setting: ICU recovery service	78	M-64%	Median 59 (IQR 54-67)	Medication changes	**Issues encountered:** Of the drugs prescribed, 135 (30%) were either new drugs or increased doses of previously prescribed drugs. Over 70% of patients were taking an increased dose of medicine or a new medicine. These new medications ranges in BNF classification. 94% of medication changes deemed appropriate by clinical team. There was a significant increase in number of patients taking regular analgesia following severe COVID-19 infection (23 (29.5%) vs 39 (50%), p< 0.001).<br>**Risk factors for problems:** Unclear<br>**Protective factors against problems:** Nil described
Morandi et al	2013	USA	General<br><br>Patients >60 years old	Single centre<br><br>Prospective observational cohort study	Hospital discharge	120	M-53%	Median 68 (IQR 64-74)	Any	**Issues encountered:** 250 PIMs and 80 AIMs identified at discharge.<br>Opiates > anticholinergics > antidepressants > Non-benzodiazepine anxiolytic > AAP > other<br>**Risk factors for problems:** Only significant for PIMS multivariable analysis: number of pre-hospital PIMs, discharge anywhere not to home, discharge from surgical service. Nil significant for AIMs.

										**Protective factors against problems:** Nil described
Morandi et al	2011	USA	General<br><br>Patients >60yrs old	Single centre<br><br>Prospective observational cohort study	Hospital discharge	120	NA	Median 68 (IQR 64-74)	Any	**Issues encountered:** 85% patients had PIM at discharge, 37% of patients had 3 or more PIMs at discharge.<br>50% of these PIMs initiated in ICU.<br>Among 103 patients with at least 1 PIM, 59% had at least 1 AIM, 59% of these initiated in ICU.<br>**Risk factors for problems:** Nil described<br>**Protective factors against problems:** Nil described
Stollings et al	2018	USA	General	Single-centre<br><br>Prospective observational cohort study	ICU-clinic: median 29 days post hospital discharge	56	M-57%	Median 48 (Range-35-57)	Any	**Issues encountered:** 22 (39%) patients had medication stopped, 18 (32%) had new medication initiated.<br>ADEs identified in 9/56 (16%) patients, ADE preventative measures in 18 (32%) patients.<br>**Risk factors for problems:** Nil described<br>**Protective factors against problems:** Nil described

Key: NA – not addressed by the paper, PIM – potentially inappropriate medication, AIM – actually inappropriate medication, ADE – adverse drug event, AAP – atypical antipsychotic, AST – acid-suppressant therapy
