# Supplementary material for: Medication-related problems in critical care survivors: a systematic review
Source: Eur J Hosp Pharm. 2023 May 4;30(5):250–6. doi: 10.1136/ejhpharm-2023-003715 (PMC10447966; doi:10.1136/ejhpharm-2023-003715)
Supplement: Supplementary data [file ejhpharm-2023-003715supp004.pdf]

S3: Table 2 GASTROPROTECTION MEDICATION

Author	Yr	Country	ICU Population	Nature	Timeline	n	Gender	Age	Results
Bell et al	2011	Canada	General<br><br>Patients ≥ 66 years, with pre-ICU medication use.	Multicentre<br><br>Retrospective observational cohort study.	Up-to 90 days post hospital discharge	16474	M-57%	Mean 75.4 (SD 5.61)	**Prescription changes at hospital discharge:** Discontinuation of medication n = 670/16474 (15.4%)<br>**Inappropriate discharge Rx?** Unclear<br>**Factors associated with continuation:** Nil described<br>**Factors associated with discontinuation:** ICU stay vs hospitalisation not including ICU admission
Blackett et al.	2021	USA	Medical<br>Cardiac<br>Cardiothoracic<br>Surgical<br>Neurological	Single centre<br><br>Retrospective, observational cohort study	Hospital discharge and first primary care visit	2467	M-59%	Highest age tertile: 18-56yrs	**Prescription changes at hospital discharge:** n=668 (27%) continued PPI at hospital discharge. 18/24 (64%) with available primary care records were continued PPI at this follow-up point.<br>I**nappropriate discharge Rx:** All identified as having no indication for long-term PPI.<br>**Factors associated with continuation:** Multivariable logistic regression model for those continued inappropriate PPI vs discontinued PPI found surgical vs medical admission, discharge to longer term care facility vs home, undergoing UGIE vs not and increased number of medications (>10 vs <8) in favour of inappropriate continuation.<br>**Factors associated with discontinuation:** Nil at multivariable modelling
Eijsbroek et al	2013	UK	General	Single Centre<br><br>Retrospective observational cohort study	ICU-clinic 3-9months post discharge	21	M-53%	Mean 64.4 (SD 13)	**Prescription changes at hospital discharge:** 2 additional patients prescribed PPI at discharge and follow-up.<br>**Inappropriate discharge Rx?** 2 patients queried continuation at follow up<br>**Factors associated with continuation:** Nil described<br>**Factors associated with discontinuation:** Nil described
Farley et al.	2013	Australia	Medical<br>Surgical<br>Cardiac	Multicentre<br><br>Retrospective observational cohort study.	Hospital discharge	387	M–58%	Mean 67.7	**Prescription changes at hospital discharge:** n = 75/190 (36%) new SUPs continued<br>n = 29/146 (20%) had pre-hospital SUP prescription changed<br>n = 11/146 (8%) pre-hospital SUP discontinued<br>**Inappropriate discharge Rx?** n = 75/190 (39%) deemed inappropriate.<br>n = 9/11 pre-hospital SUP potentially discontinued inappropriately.<br>**Factors associated with continuation:** Nil described<br>**Factors associated with discontinuation:** Nil described
Farrell et al.	2010	USA	General	Single centre<br><br>Retrospective observational cohort study	Hospital discharge	210	M=52%	Median 61	**Prescription changes at hospital discharge:** n=36/185 (19.4%) survivors discharged home on new acid-suppressing medication.<br>85.9% of survivors who were admitted with ASM were discharged on one of these medications. 31.3% discharged on different ASM to admission.<br>I**nappropriate discharge Rx:** n=35/114 (31%) survivors, not admitted on ASM, were discharged home on ASM with no indicated risk factors.

									**Factors associated with continuation:** On multivariable modelling ventilator dependent respiratory failure only significant risk for SUP use.<br>**Factors associated with discontinuation:** Nil described
Franchitti et al	2020	Switzerland	General	Single centre<br><br>Retrospective observational cohort study	Hospital discharge	140	M-69%	Median 65 (Range 17 – 92)	**Prescription changes at hospital discharge:** n = 30/130 (23.1%) new SUP continued.<br>**Inappropriate discharge Rx?** Potentially inappropriate<br>**Factors associated with continuation:** Nil described<br>**Factors associated with discontinuation:** Nil described
Hatch et al.	2010	USA	Medical<br>Surgical	Single centre<br><br>Retrospective observational cohort study.	Hospital discharge	356	M-59%	Mean 55 (SD 19)	**Prescription changes at hospital discharge:** n = 31/356 (8.7%) new SUP continued.<br>**Inappropriate discharge Rx?** n = 31/197 (15.7%) deemed inappropriate of those discharged on SUP.<br>**Factors associated with continuation:** Nil described<br>**Factors associated with discontinuation:** Nil described
Mehta et al.	2020	Canada	General<br><br>Patient age ≥ 65	Single centre<br><br>Retrospective observational cohort study.	Hospital discharge	66	M-67%	Mean 75.5 (SD 7.1)	**Prescription changes at hospital discharge:** non-naïve cohort: n = 27/31 (87%) survivors continued pre-hospital SUP.<br>Naïve cohort: n = 9/11 (82%) survivors continued new PPI<br>**Inappropriate discharge Rx?** Unclear<br>**Factors associated with continuation:** Nil described<br>**Factors associated with discontinuation:** Nil described
Murphy et al.	2008	USA	Surgical<br>Level 1 trauma	Single centre<br><br>Prospective, observational, cohort study	Hospital discharge and ICU-clinic: 4/52 post hospital discharge	248	M-63%	Median 58.0 (IQR 43 – 69.8)	**Prescription changes at hospital discharge:** n = 60/248 (24.2%) continued<br>**Inappropriate discharge Rx?** n = 3/60 (5.0%) had compelling reason for continuation, unclear n = 57/60.<br>**Factors associated with continuation:** Nil described<br>**Factors associated with discontinuation:** Nil described
Shin	2015	South Korea	General	Single centre<br><br>Retrospective observational cohort study	Hospital discharge	622	UK	UK	**Prescription changes at hospital discharge:** n=359 (57.7%) continued newly initiated PPI at hospital discharge. Percentage of continued PPI use at hospital discharge increased over the 4-year study period – 48% (2010) to 71% (2013).<br>I**nappropriate discharge Rx:** All deemed inappropriate continuation by author<br>**Factors associated with continuation:** Nil described<br>**Factors associated with discontinuation:** Nil described
Tan et al	2016	Australia	General	Multicentre<br><br>Retrospective observational cohort study	Hospital discharge	314	M-57%	60 (IQR 42-71)	**Prescription changes at hospital discharge:** n = 90/184 (48.9%) new SUP continued.<br>**Inappropriate discharge Rx?** n = 81/90 (90%) deemed inappropriate<br>**Factors associated with continuation:** Nil described<br>**Factors associated with discontinuation:** Nil described
Wohlt et al.	2007	USA	Medical<br>Surgical	Single Centre.	Hospital discharge	394	M–58%	Mean 54 (SD 19.0)	**Prescription changes at hospital discharge:** n = 96/394 (24.4%) new GAS continued.

				Retrospective observational cohort.	and ICU-clinic: 3-months post hospital discharge				n = 3/394 (0.76%) discontinued GAS.<br>n = 32/55 (58.2%) follow-up patients ongoing GAS prescription.<br>**Inappropriate discharge Rx?** Yes, all continuations/discontinuations deemed inappropriate.<br>**Factors associated with continuation:** Nil described<br>**Factors associated with discontinuation:** Nil described

Key: AST – acid-suppressant therapy, GAS – gastric-acid suppressant, PPI – proton-pump inhibitor, SUP – stress ulcer prophylaxis
