# Supplementary material for: Medication-related problems in critical care survivors: a systematic review
Source: Eur J Hosp Pharm. 2023 May 4;30(5):250–6. doi: 10.1136/ejhpharm-2023-003715 (PMC10447966; doi:10.1136/ejhpharm-2023-003715)
Supplement: Supplementary data [file ejhpharm-2023-003715supp005.pdf]

S3: Table 3 Psychotropic medication data extraction table

Author	Yr	Country	ICU Population	Nature	Timeline	n	Gender	Age	Medication type	Results
Coe et al	2020	USA	General	Multi-centre.<br><br>Retrospective observational cohort study.	Post hospital discharge: up to 180 days post discharge	134,999	M–96%	Mean 66.5 (SD 11)	**Antipsychotics**	**Prescription changes at hospital discharge:** Antipsychotic continued: total population n = 3278 (2.43%),<br>ICU plus sepsis cohort = 361 (3.6%), ICU minus sepsis cohort = 2917 (2.3%)<br>**Inappropriate discharge Rx?** Unclear<br>**Factors associated with continuation:** Diagnosis of sepsis at ICU admission<br>**Factors associated with discontinuation:** Nil described
Dixit et al.	2021	USA	General	Multicentre<br><br>Retrospective observational cohort study	Hospital discharge	300	M-65%	Median 69 (IQR 55-78)	**Antipsychotics**	**Prescription changes at hospital discharge:** 61% of patients continued antipsychotic at hospital discharge.<br>**Inappropriate discharge Rx:** Unclear, some medications restarted post-ICU discharge.<br>**Factors associated with continuation:** Discharge from 'mixed ICU' compared to medical and surgical ICU.<br>**Factors associated with discontinuation:** Prolonged duration of antipsychotic prescription, longer ICU and hospital LoS reduced likelihood of continuation at hospital discharge. Antipsychotic discontinued in ICU had significantly lower OR for continuation of antipsychotic at hospital discharge.
Farrokh et al.	2017	USA	Medical<br>Surgical<br>Cardiac<br>Neurosurgical	Single centre<br><br>Retrospective observational, pseudo-randomised study.	Hospital discharge	100	M-76%	Median 65 (range 21-95)	**Atypical antipsychotics**	**Prescription changes at hospital discharge:** Medication continued: n = 23 (23%)<br>**Inappropriate discharge Rx?** Unclear. CAM-ICU rarely documented<br>**Factors associated with continuation:** Admission to SICU (30%) > CICU > CTICU > MICU (19%)<br>**Factors associated with discontinuation:** Nil described
Flurie et al.	2015	USA	Medical	Single centre.<br><br>Retrospective observational cohort study.	Hospital discharge	87	M-52%	-	**Antipsychotics**	**Prescription changes at hospital discharge:** Medication continued: n = 9/87 (10.3%)<br>**Inappropriate discharge Rx?** Unclear<br>**Factors associated with continuation:** Higher proportion discharged on medication if continued from ICU to ward, n = 9/23 (39%).<br>**Factors associated with discontinuation:** Nil described

Gilbert at al.	2017	USA	Medical<br>Surgical<br>Cardiac<br>Neurosurgical	Single centre<br><br>Retrospective observational cohort study.	Hospital discharge	161	M–35%	50	**Neuroleptics**	**Prescription changes at hospital discharge:**<br>Medication continued: n = 54 (34%)<br>**Inappropriate discharge Rx?** Study deemed all inappropriate because no documented physician notes for medication use in chronic management<br>**Factors associated with continuation:** Multiple neuroleptics or trazadone during ICU stay<br>**Factors associated with discontinuation:** Prescription of haloperidol in ICU
Jasiak et al	2012	USA	Medical	Single centre<br><br>Retrospective observational cohort study	Hospital discharge	80	-	Mean 59 (SD 17.2)	**Antipsychotic**	**Prescription changes at hospital discharge:**<br>Medication continued: n = 20/59 survivors (33.9%)<br>**Inappropriate discharge Rx?** Unclear<br>**Factors associated with continuation:** “Increased” ICU LOS, “increased” hospital LOS, final CAM-ICU positive.<br>**Factors associated with discontinuation:** Nil described
Karamchandani et al.	2018	USA	Surgical<br>Medical<br>Cardiovascular<br>Neurosurgical	Single centre<br><br>Retrospective observational cohort study.	Hospital discharge	346	M–66%	Median 59.9 (Range 18 – 99)	**Atypical antipsychotic**	**Prescription changes at hospital discharge:**<br>Medication continued: n = 174/314 survivors (55%)<br>**Inappropriate discharge Rx?** Unclear<br>**Factors associated with continuation:** Continued care facility at DC, male, short hospital LOS, longer ICU LOS<br>**Factors associated with discontinuation:** Nil described
Kram et al.	2015	USA	Medical<br>Surgical<br>Cardiac<br>Neurosurgical<br>Cardiothoracic surgical	Single centre<br><br>Retrospective observational cohort study.	Hospital discharge	133	M–68%	Median 61.5 (IQR 45 - 71)	**Atypical antipsychotic**	**Prescription changes at hospital discharge:**<br>Medication continued: n = 38/133 (28.6%) survivors<br>**Inappropriate discharge Rx?** Potentially inappropriate, 26/38 had no ongoing reason for continuation<br>**Factors associated with continuation:** Discharge to long term care facility, diagnosis TBI<br>**Factors associated with discontinuation:** Nil described
Lambert et al.	2021	Belgium	General	Single centre<br><br>Retrospective observational cohort study	Hospital discharge	196	M-78%	Median 67 (IQR 53-76)	**Antipsychotic**	**Prescription changes at hospital discharge:** following ICU initiation of medication, n=38 (19.4%) continued antipsychotic at hospital discharge.<br>25/41 (61%) patients discharged on antipsychotic had indication for continued antipsychotic discussed on discharge letter.<br>**Inappropriate discharge Rx:** Unclear<br>**Factors associated with continuation:** On multivariable logistic regression modelling – admission to medical ICU or receiving quetiapine increased risk of continued antipsychotic.<br>**Factors associated with discontinuation:** Nil described

Levine et al.	2019	USA	Medical<br>Surgical	Single centre<br><br>Retrospective observational cohort study	Hospital discharge	124	Medical M-68%<br><br>Surgical M-61%	Age >60yrs: Medical -62%<br><br>Surgical -46%	**Antipsychotics**	**Prescription changes at hospital discharge:** n=29/78 (37.2%) medical ICU patients and n=25/46 (54.3%) surgical ICU patients continued antipsychotics at hospital discharge.<br>**Inappropriate discharge Rx:** Unclear.<br>**Factors associated with continuation:** Medical ICU patients – higher risk on multivariable analysis if history of pre-existing dementia (OR = 10, 95% CI 1.11 – 90.5), longer hospital stay and discharge to skilled nursing facility. Surgical ICU patients – severe TBI and initiation on quetiapine > olanzapine.<br>**Factors associated with discontinuation:** Discharge to home in both medical and surgical populations.
Marshall et al.	2016	USA	Medical	Single centre<br><br>Retrospective observational cohort study	Hospital discharge	3119	F-40%	Mean 66	**Antipsychotic**	**Prescription changes at hospital discharge:** n=642/3119 (21%) continued on newly initiated antipsychotics at hospital discharge.<br>**Inappropriate discharge Rx:** Unclear<br>**Factors associated with continuation:** Multivariable analysis higher risk if discharged to facility other than home (OR = 2.4, 95% CI 1.9 – 3.1), admission from ED (OR = 1.4, 95% CI 1.2 – 1.7). Prescription of quetiapine>olanzapine>haloperidol.<br>**Factors associated with discontinuation:** Nil described.
Rowe et al.	2015	USA	Trauma-surgical<br>Neurosurgical	Single centre<br><br>Retrospective observational cohort study	Hospital discharge	341	F-30%	Median 50-55	**Antipsychotic**	**Prescription changes at hospital discharge:** n=82/341 (24%) prescribed new antipsychotic medication. Majority (81.8%) for quetiapine.<br>**Inappropriate discharge Rx?** N=52/82 (67.1%) deemed inappropriate continuation as described in notes – no standardised measure of delirium used.<br>**Factors associated with continuation:** Higher APACHE score at admission to ICU, longer ICU LoS (14 days +/-14 vs 4 days +/- 11), longer hospital LoS, received higher morphine equivalents during admission (1254mg +/- 4410.5 vs 198.5mg +/- 1094) and more benzodiazepine usage days during admission (14days +/- 12 vs 3 +/- 11)<br>**Factors associated with discontinuation:** Nil described
Tomichek et al	2016	USA	Medical<br>Surgical	Single centre<br><br>Prospective observational cohort study	Hospital discharge	500	F-45%	Median 59 (IQR 49-69)	**Antipsychotic**	**Prescription changes at hospital discharge:** Medication continued: n= 42/172 (24.4%) of survivors treated with AP during ICU<br>**Inappropriate discharge Rx?** Potentially inappropriate in 28/42 with documented normal mental status, potentially appropriate in 7/42 with ongoing delirium.

										**Factors associated with continuation:** Received AAP and not haloperidol during hospital stay<br>**Factors associated with discontinuation:** Nil described
Wunsch et al.	2014.	Denmark	Medical	Multi-centre.<br><br>Retrospective observational cohort study.	Post hospital discharge: filled prescription up to 1-year post discharge	24179	M-62%	Median age range: 65-79 = 10020	**Psychiatric medications**	**Prescription changes at hospital discharge:**<br>Medication continued:<br>At 3-month: n = 1261/9912 (12.7%) of survivors<br>At 12-month: n = 101/6485 (1.6%) of survivors. 12-month risk of continuation similar to non-ICU population.<br>**Inappropriate discharge Rx?** Unclear<br>**Factors associated with continuation:** ICU admission compared to general hospital admission.<br>**Factors associated with discontinuation:** Nil described
