# Supplementary material for: Medication-related problems in critical care survivors: a systematic review
Source: Eur J Hosp Pharm. 2023 May 4;30(5):250–6. doi: 10.1136/ejhpharm-2023-003715 (PMC10447966; doi:10.1136/ejhpharm-2023-003715)
Supplement: Supplementary data [file ejhpharm-2023-003715supp006.pdf]

S3_Table 4 ANALGESIA MEDICATION

Author	Yr	Country	ICU Population	Nature	Timeline	n	Gender	Age	Analgesia type	Results
Academia et al	2020	USA	Medical Cardiac	Single Centre<br><br>Retrospective observational cohort study.	Hospital discharge	71	M-62%	Mean 56.7 (SD 13.5)	**OPIATE**	**Prescription changes at hospital discharge:** n= 32/71 (45%) new opiate prescribed. Oxycodone most frequent oral opiate prescribed.<br>**Inappropriate discharge Rx?** 36.7% of new opioid prescription inappropriate based on pre-discharge analgesia requirements.<br>**Factors associated with continuation:** CICU admission.<br>**Factors associated with discontinuation:** Shorter duration (median 4 vs 9.8 days) inpatient opiate use.
Eijsbroek et al	2013	UK	General	Single centre<br><br>Retrospective observational cohort study.	ICU-clinic: 3- to 9-months post discharge	21	M-52%	Mean 64.4 (SD 13)	**ALL**	**Prescription changes at hospital discharge:** n = 16/21 (76%) new analgesia. n = 13/17 new analgesic drugs non-opioid<br>**Inappropriate discharge Rx?** Patients and carers raised concerns about unnecessary prescribing of analgesics and ineffective pain control.<br>**Factors associated with continuation:** Nil described<br>**Factors associated with discontinuation:** Nil described
Karamchandani et al.	2019	USA	Surgical Veteran Hospital	Single centre<br><br>Retrospective observational cohort study	3-months post hospital discharge and annually for 3-years	193328	M-97%	Mean 62.5 (9.0) and 66.8 (10.0)	**OPIATE**	**Prescription changes at hospital discharge:** n=7729 developed new, persistent opioid use (defined as use at 3-months). Annual decline of 6% per year in persistent opioid use.<br>**Inappropriate discharge Rx?** Unclear<br>**Factors associated with continuation:** Younger age (mean 62.5 vs 66.8 years) and greater prevalence of alcohol and substance use disorder.<br>**Factors associated with discontinuation:** Nil described
Kranchevich et al.	2021	USA	Medical, surgical, cardiac.	Multicentre<br><br>Retrospective observational cohort study.	Hospital discharge and post-hospital prescriptions at 3-, 6- and 12-months	342	M-64%	Mean 55.8	**OPIATE**	**Prescription changes at hospital discharge:** n = 164 (47.1%) new opiates prescribed at discharge.<br>5% of entire cohort had ≥ opiate fills in 12-months post discharge, significantly higher incident if discharged from hospital with opiate.<br>**Inappropriate discharge Rx?** Unclear<br>**Factors associated with continuation:** history of illicit drug use, longer non-ICU LOS, ICU admission diagnosis of respiratory, surgical, trauma or malignancy.<br>**Factors associated with discontinuation:** older age

MacTavish et al.	2020	UK	Medical, surgical	Multicentre<br><br>Prospective observational cohort study	ICU-clinic: 4- to 12-weeks post hospital discharge	183	M-56%	Median 58 (IQR 50-65)	**ALL**	**Prescription changes at hospital discharge:** n = 50/183 (27%) new analgesia. New regular opioid use 30/183 (16%)<br>**Inappropriate discharge Rx?** Unclear<br>**Factors associated with continuation:** Nil described<br>**Factors associated with discontinuation:** Nil described
MacTavish et al	2021	UK	General (COVID-19 survivors)	Multicentre<br><br>Prospective observational cohort study	3-7 months post hospital discharge (ICU clinic)	78	m-64$	Median 59 (IQR 54-67)	**Medication changes**	**Prescription changes at hospital discharge:** There was a significant increase in the number of patients taking regular analgesia following severe COVID-19 infection (23 (29.5%) vs 39 (50%), p<0.001). Of those patients who were receiving either no pain medication or non-opioid pain relief (WHO ladder step 1) before critical care, 8 (10%) were receiving weak or strong opioids (WHO ladder step 2 or 3).<br>**Inappropriate: discharge RX**: not clear<br>**Factors associated with continuation:** Nil described<br>**Factors associated with discontinuation:** Nil described
Tollinche et al	2022	USA	General ICU within Cancer Centre	Single centre<br><br>Retrospective observational cohort study	Hospital discharge	848	M=56%	Median 64(IQR 52-72)	**OPIATE**	**Prescription changes at hospital discharge:** n=346 (40.8%) discharged with new opiate prescription.<br>I**nappropriate discharge Rx:** Unclear<br>**Factors associated with continuation:** Multivariable modelling found pre-admission benzodiazepine use (OR 3.01, 95% CI 1.41 – 6.45), diagnosis of sepsis at ICU admission (OR 12.99, 95% CI 8.58 – 19.67), and continuous opioid infusion >4h (OR 3.06, 95% CI 1.98 – 4.73) the highest factors linked to continued opioid prescription.<br>**Factors associated with discontinuation:** Univariable analysis – use of propofol infusion, opioids and benzodiazepines during ICU stay associated with decreased odds of new opioid at discharge.
Wang et al	2018	Canada	General<br><br>Patients >65 and chronic opioid pre-ICU admission	Multicentre<br><br>Retrospective observational cohort study.	Post-hospital discharge: filled prescription at 80-days	28570	F-60%	Mean 76.7 (SD 7.1)	**OPIATE**	**Prescription changes at hospital discharge:** n = 12403/19584 (63.3%) survivors had filled an opiate prescription in 180-days post hospital discharge.<br>n = 1841/19584 (9.4%) had not filled any opiate prescription in 180-days post hospital discharge.<br>22.0% of patients had higher MEQ dose cf. pre-hospitalization dose, 19.8% were receiving the same dose, and 21.5% a lower dose.<br>**Inappropriate discharge Rx?** Unclear<br>**Factors associated with continuation:** COPD, medical patient, fentanyl as primary opioid at admission, concurrent benzodiazepine use at admission.

										**Factors associated with discontinuation:** Increased age, dementia, MV, tracheostomy, dialysis and codeine or oxycodone as primary opioid at admission.
Witcraft et al.	2021	USA	Medical	Single centre.<br><br>Retrospective observational cohort study	Hospital discharge	66	M-53%	Mean 58.7 (SD 15.5)	**OPIATE**	**Prescription changes at hospital discharge:** n = 21 (31.8%) new analgesia<br>**Inappropriate discharge Rx?** Possibly - median pain score at hospital DC 0 (IQR 0-5)<br>**Factors associated with continuation:** Higher rate intubation and cumulative dose (opioid fentanyl equivalent dose)<br>**Factors associated with discontinuation:** Nil described
Wunsch et al.	2020	Canada	General	Multicentre.<br><br>Retrospective observational cohort study.	Post hospital discharge: filled prescription at day-7 and 1-year	25085	M-58%	Mean 61.7 (SD 17.9)	**OPIATE**	**Prescription changes at hospital discharge:** n = 5007 (20%) new opioid prescription filled at day 7.<br>'Persistent' opioid use at 1-year ranges from 2.6 - 4.9% depending on definition of 'persistent' used.<br>**Inappropriate discharge Rx?** Unclear<br>**Factors associated with continuation:** Surgical patient higher rate of opiate continuation compared to medical patient for both 7-day and 1-year prescription filling.<br>**Factors associated with discontinuation:** Older patient, greater number comorbidities, longer ICU LOS.
von Oelreich et al	2021	Sweden	General	Multicentre<br><br>Retrospective observational cohort	Up to 24 months post hospital discharge	204402	M=59%	Median 63 (IQR 46-73)	**OPIATE**	**Prescription changes at hospital discharge:** Both populations of opioid naïve and non-opioid naïve patients had initial peak of mean opioid consumption in first quarter following ICU admission. This declined over subsequent 24 months but not returning to baseline. 22,138 developed chronic opioid use following critical care (defined as at least one prescription days 1-90 and days 91-180 post discharge)<br>I**nappropriate discharge Rx:** Unclear<br>**Factors associated with continuation:** Multivariable analysis – higher risk of chronic opioid use: Pre-ICU opioid use (OR 10.31, 95% CI 9.96 – 10.67), acute care surgery (OR 1.40, 95% CI 1.24 – 1.37).<br>**Factors associated with discontinuation:** Nil described.
Yaffe et al	2021	Canada	General	Single centre<br><br>Retrospective observational cohort	Hospital discharge and annually up to 4-years post discharge	2595	M=60%	Median 46 (IQR 21)	**OPIATE**	**Prescription changes at hospital discharge:** Majority of patients (77%) were non-users of opiates at baseline. The number of non-opiate users in the whole population increased at hospital discharge (88%) and further at 12- and 36-month timepoints (91% and 94% respectively). This correlated to a reduction in intermittent (17% pre-admission – 2.6% at 48-months) and chronic opiate use (6.2% pre-admission – 1.8% at 48-months)

										**Inappropriate discharge Rx:** Unclear<br>**Factors associated with continuation:** Chronic opiate use pre-admission and longer ICU LOS.<br>**Factors associated with discontinuation:** Non-opiate user at pre-admission timepoint.
