# Supplementary material for: Medication-related problems in critical care survivors: a systematic review
Source: Eur J Hosp Pharm. 2023 May 4;30(5):250–6. doi: 10.1136/ejhpharm-2023-003715 (PMC10447966; doi:10.1136/ejhpharm-2023-003715)
Supplement: Supplementary data [file ejhpharm-2023-003715supp007.pdf]

S3: Table 5 CARDIAC MEDICATIONS

Author	Yr	Country	ICU Population	Nature	Timeline	n	Gender	Age	Medication type	Cardiac
Bell et al	2011	Canada	General<br><br>Patients ≥ 66 years, with pre-ICU medication use.	Multicentre<br><br>Retrospective observational cohort study.	Up-to 90 days post hospital discharge	16474	M-57%	Mean 75.4 (SD 5.61)	Statin<br><br>Antiplatelet<br><br>Anticoagulant	**Prescription changes at hospital discharge:** n = 1484/10138 (14.6%) of patients on pre- ICU statins were discontinued.<br><br>n = 552/2423 (22.8%) patients on pre-ICU antiplatelet/anticoagulant were discontinued.<br><br>Higher OR of discontinuation in ICU population compared to non-ICU hospitalized cohort<br><br>**Inappropriate discharge Rx?** Yes, results analysed as unintentional discontinuation<br><br>**Factors associated with continuation: Nil** described<br><br>**Factors associated with discontinuation:** Nil described
Choon et al	2021	UK	General<br><br>Patients with AKI for KRT	Single centre<br><br>Retrospective observational cohort study	Hospital discharge	91	M-55%	Median 61 (IQR 47-71)	Antihypertensive<br>Statin<br>Diuretics<br>Anti-diabetic	**Prescription changes at hospital discharge:** Higher frequency of changes in not restarting pre-hospital medication: n=15/35 patients not restarted RAASi at discharge, n=7/20 patients not restarted antidiabetic drugs, n=8/25 patients not restarted a diuretic and n=23/39 not restarted a statin.<br>**Inappropriate discharge Rx:** Unclear<br>**Factors associated with continuation:** Nil described<br>**Factors associated with discontinuation:** Nil described
Coe et al	2020	USA	General/VA Hospitals<br><br>Statin prescription fill within 180days pre-hospital	Multicentre<br><br>Retrospective observational cohort study.	Post hospital discharge (up to 180 days post discharge	82242	M–97%	Mean 67.9 (SD 9.5)	Statin	**Prescription changes at hospital discharge:** n = 899/5939 (15.1%) with sepsis diagnosis did not fill a statin prescription following discharge.<br><br>n = 7611/76303 (10.0%) without sepsis diagnosis did not fill a statin prescription following discharge.<br><br>**Inappropriate discharge Rx?** Unclear<br><br>**Factors associated with continuation:** Adjusted to hospital performance, worse performing hospitals had 11% higher odds of discontinuation.<br><br>**Factors associated with discontinuation:** Admission without sepsis had OR 0.83 (0.77, 0.90) of statin discontinuation
Rizvi et al	2019	USA	General	Single centre<br><br>Retrospective observational cohort study	Hospital discharge	1010	M-57%	Mean 63.6 (SD 14.8)	Midodrine	**Prescription changes at hospital discharge:** n = 311/909 (34%) survivors continued medication.<br><br>Discharge from hospital on midodrine had 1.6-fold higher risk of death in following year<br><br>**Inappropriate discharge Rx?** Potentially inappropriate, 50% of survivors also prescribed antihypertensives.

										**Factors associated with continuation:** Congestive heart failure<br><br>**Factors associated with discontinuation:** hypertension, use of IMV, surgical ICU admission, pharmacy intervention (small 10% sub-population reviewed)
